# Editorial: Advanced electrochemical energy devices

**DOI:** 10.3389/fchem.2022.1121482

**Published:** 2023-01-04

**Authors:** Tao Wei, Cheng Sun, Sijia Wang, Mengting Wang, Daifen Chen

**Affiliations:** School of Energy and Power, Jiangsu University of Science and Technology, Zhenjiang, China

**Keywords:** electrochemical energy devices, supercapacitors, fuel cells, potassium-ion batteries, solid oxide electrolysis cell

## 1 Foreword

The ever-increasing environmental issues and energy crisis have summoned up the carbon neutral strategy all over the world, thus promoting the development of new energy conversion technologies, such as wind, solar, fuel cells, as well as new energy storage technologies, especially electrochemical energy devices, among the various technologies, Supercapacitors (SCs) (Wei et al., 2017), Li/K/Zn/Na/Mg ion/air batteries (Wei et al., 2020), and fuel cells (Wei et al., 2014) as advanced next-generation power sources have evoked a plethora of research owing to their high energy density, flexibility of scale and environmentally friendly features.

For the purpose of accelerating the development of electrochemical energy conversion and storage industry, a Research Topic of “Advanced Electrochemical Energy Devices” is proposed by the journal of Frontiers in Chemistry. Experts and researchers from many famous universities were invited to share their prospects or progress in this field. This Research Topic includes 4 papers, including 3 research papers and a review, which represents the current hot research directions in advanced electrochemical energy devices and the authors have given their insightful opinions about these technologies.

## 2 Topic A: Graphene hydrogels used in SCs

Supercapacitor (also called pseudocapacitor) is a new type of energy storage device developed in recent years. It has the characteristics of high-power density, long service life, and rapid charge and discharge which can be used in self-powered equipment and electric vehicles. Graphene and its derivatives have been studied extensively in SCs for their high specific surface areas and conductivities. With a fully utilized surface area of one single-layer graphene, a theoretical specific capacitance of 550 F g^−1^ can be obtained (El-Kady et al., 2012). However, the graphene sheets tend to re-stack during fabrication. Graphene hydrogels and aerogels with self-assembled 2D graphene sheets into 3D framework seems to be an efficient way to solve the issue of stacking (De et al., 2017; Kou et al., 2015). Ju et al. presented a facile two-step hydrothermal method to achieve a functionalized graphene oxide hydrogels as binder-free electrodes, the assembled symmetric SC delivered a high specific energy of 39 Wh kg^−1^ at a specific power of 749 W kg^−1^, while still maintaining 88.09% of its initial capacitance after 10,000 cycles.

## 3 Topic B: Flexible potassium-ion batteries (PIBs)

Potassium is much more abundant in the Earth’s crust compared to lithium (.0017 wt% for Li and 1.5 wt% for K), thus the prices of potassium precursors are much cheaper than lithium precursors which was used to produce the corresponding metal (Min et al., 2021). Therefore, PIBs are considered another competitive alternative for lithium-ion batteries (Wei et al., Forthcoming 2022). What’s more, flexible devices such as wearable devices and portable soft electronic equipment are urgently needed by the modern society, it should be a huge market for the flexible PIBs, however, the applications of flexible PIBs are still scarce. Li et al. systematically reviewed the recent progresses of carbon-based flexible anodes for PIBs.

## 4 Topic C: Solid oxide electrolysis cell (SOECs)

SOEC is an attractive device that can produce synthesis gas (a mixture of H_2_ and CO) from H_2_O and CO_2_ from excess renewable power, which operates through a reverse reaction of solid oxide fuel cell (SOFC) (Zheng et al., 2017). However, SOEC needs to work in high temperature and humidity environment, whereas, the conventional Ni-YSZ electrode suffers from the agglomeration of Ni particles (Yue and Irvine, 2012). In order to overcome this disadvantage, some perovskite-based oxides were proposed. Thus, Zhen et al. proposed Ni/Ti co-doped Sr_1.95_Fe_1.2_Ni_0.1_Ti_0.2_Mo_0.5_O_6-δ_ double perovskite oxides and used it for effective CO_2_ reduction, the cell exhibits excellent stability at 1.4 V after 100 h.

## 5 Perspective

This “Advanced Electrochemical Energy Devices” Research Topic introduces some latest development in electrochemical energy devices, which is believed to provide representative progresses in this area. For the purpose of achieving the carbon neutral society, much work still should be done in future. We sincerely thank all the authors, reviewers, and the editorial team of Frontiers in Chemistry for their hard works.

## References

[B1] De B. Kuila T. Kim N. H. Lee J. H. (2017). Carbon dot stabilized copper sulphide nanoparticles decorated graphene oxide hydrogel for high performance asymmetric supercapacitor. Carbon 122, 247–257. 10.1016/j.carbon.2017.06.076

[B2] El-Kady M. F. Strong V. Dubin S. Kaner R. B. (2012). Laser scribing of high-performance and flexible graphene-based electrochemical capacitors. Science 335, 1326–1330. 10.1126/science.1216744 22422977

[B3] Kou L. Liu Z. Huang T. Zheng B. Tian Z. Deng Z. (2015). Wet-spun, porous, orientational graphene hydrogel films for high-performance supercapacitor electrodes. Nanoscale 7, 4080–4087. 10.1039/C4NR07038K 25660705

[B4] Min X. Xiao J. Fang M. Wang W. Zhao Y. Liu Y. (2021). Potassium-ion batteries: Outlook on present and future technologies. Energy Environ. Sci. 14, 2186–2243. 10.1039/D0EE02917C

[B5] Wei T. Lu J. Zhang P. Yang G. Sun C. Zhou Y. (Forthcoming 2022). Metal–organic framework-derived Co_3_O_4_ modified nickel foam-based dendrite-free anode for robust lithium metal batteries. Chin. Chem. Lett., 107947. 10.1016/j.cclet.2022.107947

[B6] Wei T. Zhang Z. Wang Z. Zhang Q. Ye Y. S. Lu J. H. (2020). Ultrathin solid composite electrolyte based on Li_6.4_La_3_Zr_1.4_Ta_0.6_O_12_/PVDF-HFP/LiTFSI/Succinonitrile for high-performance solid-state lithium metal batteries. ACS Appl. Energy Mat. 3, 9428–9435. 10.1021/acsaem.0c01872

[B7] Wei T. Zhang Mi. Wu P. Tang Y. J. Li S. L. Shen F. C. (2017). POM-based metal-organic framework/reduced graphene oxide nanocomposites with hybrid behavior of battery-supercapacitor for superior lithium storage. Nano Energy 34, 205–214. 10.1016/j.nanoen.2017.02.028

[B8] Wei T. Zhou X. Hu Q. Gao Q. Han D. Lv X. (2014). A high power density solid oxide fuel cell based on nano-structured La_0.8_Sr_0.2_Cr_0.5_Fe_0.5_O_3-δ_ anode. Electrochim. Acta 148, 33–38. 10.1016/j.electacta.2014.10.020

[B9] Yue X. Irvine J. T. S. (2012). Alternative cathode material for CO_2_ reduction by high temperature solid oxide electrolysis cells. J. Electrochem. Soc. 159, F442–F448. 10.1149/2.040208jes

[B10] Zheng Y. Wang J. Yu B. Zhang W. Chen J. Qiao J. (2017). A review of high temperature Co-electrolysis of H_2_O and CO_2_ to produce sustainable fuels using solid oxide electrolysis cells (SOECs): Advanced materials and technology. Chem. Soc. Rev. 46, 1427–1463. 10.1039/c6cs00403b 28165079

